# Sensory processing in children and adolescents shortly after the onset of anorexia nervosa: a pilot study

**DOI:** 10.1186/s13030-022-00256-z

**Published:** 2022-12-12

**Authors:** Tasuku Kitajima, Ryoko Otani, Takeshi Inoue, Naho Matsushima, Naoki Matsubara, Ryoichi Sakuta

**Affiliations:** Center for Child Development and Psychosomatic Medicine, Dokkyo Medical University Saitama Medical Center, Koshigaya, Saitama, Japan

**Keywords:** Sensory profile, Hypersensitivity, Sensory processing, Anorexia nervosa, Adolescents

## Abstract

**Background:**

Alterations in sensory processing, such as vision, taste, and interoceptive sensation, have been reported in adult anorexia nervosa (AN). Whether these symptoms are traits, states, or “scars” due to chronic starvation has not been fully established. Based on the hypothesis that alterations in sensory processing also occur in adolescent AN in the early stages of the disease, the present study was conducted using both self-administered and parent-administered sensory processing questionnaires.

**Methods:**

Children and adolescents with anorexia nervosa treated at a single tertiary eating disorder treatment center in Japan (AN group) and female junior high school students attending a public junior high school in Saitama Prefecture, Japan (healthy control group: HC group) were included in the study. The Sensory Profile (SP) and Adult/Adolescent Sensory Profile (AASP) were administered to the participants and their caregivers. In addition, we collected demographic data and administered the Children’s Eating Attitude Test and Autism-Spectrum Quotient Children’s version.

**Results:**

Seventeen children and adolescents were enrolled in the AN group, and 63 were enrolled in the HC group. There was no statistically significant difference between the AN and HC groups in the quadrant scores of the AASP. In the SP, the Sensory Avoiding score and the Emotional/Social response score were higher in the AN group than in the HC group.

**Conclusion:**

From the parents’ point of view, the patient avoids unexpected sensory stimuli, but the patients are unaware of their own avoiding behavior in the early stages of the disease. The results suggest that sensory sensitivity in AN may be a “scar” symptom due to chronic starvation and a state symptom. Longitudinal studies from shortly after the onset with larger sample sizes are needed to gain insight into the dynamic relation between sensory processing and eating disorder pathology.

## Background

Anorexia nervosa (AN) is a common psychiatric disorder that presents with a variety of psychosomatic symptoms and abnormal eating behaviors [1]. AN was believed to be caused by psychosocial factors, but in recent years, evidence has accumulated that AN is a disease associated with biological changes that develop due to a combination of genetic and environmental factors [2]. However, the biological pathogenesis that can explain the symptoms in a unified manner has yet to be elucidated. In addition, it is not yet possible to distinguish whether the various symptoms of AN are traits that exist before the onset of the disease, states that reflect the condition after the onset of the disease, or “scars” due to chronic starvation [3]. Distinguishing these symptoms is important for investigating factors that contribute to the development and maintenance of AN and for developing new treatments, but longitudinal studies are difficult to perform because of their cost and the high levels of attrition among individuals with AN.

Body image disturbance is one of the central symptoms of AN. Several studies have reported the presence of multisensory impairment in AN, which has been suggested to lead to body image disturbance [4–6].

There are several reports on the subjective sensory experience of patients with AN in which self-administered tests were used as the method to assess sensory impairment [7–9]. It has been suggested that multisensory impairment in AN is a trait symptom regardless of its nutritional status because abnormal sensory processing is observed in AN in both the acute and remission phases [9]. If multisensory impairment in AN is a trait symptom, it should be observed from the early onset of AN. However, these studies included adults and adolescents more than one year after the onset of AN, and it is not clear whether the sensory processing problems existed before the onset or emerged after the onset [7–9].

To assess sensory impairment, several physiological examinations are used in addition to questionnaire-based assessment [10–21]. Questionnaires are easy to administer, but they do not capture objective changes in sensory processing. Physiological examinations provide objective indicators but are often difficult to perform clinically because of the complexity of the examinations, and examinations of responses to smell and pain are often invasive for patients with AN. To develop a treatment based on the state of multisensory impairment, it is desirable to use a simpler method of assessment.

Although multisensory impairment has been investigated using subjective self-administered questionnaires [4–6], there is no study based on the perspective of others, such as parents and caregivers. In recent years, it has been found that the involvement of family members in the treatment of child and adolescent AN improves the prognosis [22]. It is clinically meaningful to understand not only the subjective sensory experience of the patient but also the way in which the family views it.

In the present study, we aimed to verify the hypotheses that “abnormalities in subjective sensory experiences suggestive of multisensory impairment can be observed even in children and adolescents with early-stage AN” and that “multisensory impairment in children and adolescents with AN can be accurately captured through the assessments from the perspective of family members.”

## Methods

### Participants

This study was conducted among children and adolescents with AN requiring inpatient treatment and their caregivers (the AN group) and age-matched healthy controls and their caregivers (the HC group).

The AN group consisted of children aged 11–18 years who first visited the Child Development and Psychosomatic Medicine Center at Dokkyo Medical University Saitama Medical Center from June 23, 2019, to December 31, 2020. They and their parents gave consent and assent to participate in the study. They were diagnosed according to the DSM-5, and two specialists in child psychiatry certified by the Japanese Society for Child and Adolescent Psychiatry, the Japanese Society of Pediatric Psychiatry and Neurology and the Japanese Society of Psychosomatic Pediatrics confirmed the diagnosis. All participants in the AN group were monitored for more than one year, and their diagnoses, all AN restrictive type, were confirmed to be correct.

As the HC group, female students who attended a public junior high school in Saitama Prefecture, Japan, and their parents or caregivers were recruited. It was confirmed by a preliminary questionnaire to the parents that the participants were not in classes for special needs children and that they did not have a psychiatric or neurodevelopmental disorder.

### Procedure

The children in the AN group completed the questionnaire test battery at the time of their first outpatient visit. Demographic data were collected at the time of the test, and height and weight were extracted from the medical records as measured on the day of the test or within a week. For the control group, information on age, sex, and self-reported height and weight were collected when the questionnaire was administered. The demographic and background data are presented in Table 1.

**Table 1:** Demographic and background data

	HC group (*n* = 63)	AN group (*n* = 17)	*P*	*d, r*
Age (months)	162 ± 8	164 ± 19	0.29	*d* = 0.18
Height (cm)	154.1 ± 6.4	150.3 ± 9.2	0.05	*d* = 0.54
Weight (kg)	45.9 ± 6.4	31.0 ± 6.5	**< 0.01**	*d* = 2.31
BMI (kg/m^2^)	19.3 ± 2.1	13.6 ± 1.8	**< 0.01**	*d* = 2.76
BMI-SDS	-0.2 ± 0.9	-3.8 ± 1.6	**< 0.01**	*d* = 3.36
Duration of illness (months)		8 ± 5		
ChEAT26 total score	8(0–39)	25(3–51)	**< 0.01**	*r* = 0.45
Preoccupation with thinness	1(0–13)	5(0–15)	0.34	*r* = 0.11
Food preoccupation	0(0–6)	3(0–14)	**< 0.01**	*r* = 0.47
Dieting	3(0–13)	7(3–21)	**< 0.01**	*r* = 0.56
Social pressure to eat	0(0–10)	6(0–11)	**< 0.01**	*r* = 0.53
Purging	0(0–2)	0(0–3)	**< 0.01**	*r* = 0.43
Total AQC score	14(5–38)	19(11–34)	0.07	*r* = 0.20
Social skills	4(0–10)	4(0–9)	1.00	*r* = 0.00
Attention switching	3(0–9)	4(1–8)	0.06	*r* = 0.21
Attention to detail	3(1–7)	5(0–7)	**0.01**	*r* = 0.29
Communication	2(0–8)	3(0–6)	0.14	*r* = 0.16
Imagination	3(0–9)	3(1–6)	0.98	*r* = 0.00

Statistics reported are means (± standard deviation) for age, height, weight, BMI, BMI-SDS and duration of illness. Due to nonnormal distributions, statistics reported are medians followed by the range in parentheses for ChEAT26 and AQC scores. Cohen’s d (*d*) was used for the effect size in the t tests and Pearson product-moment correlation coefficient (*r*) for the Mann Whitney U test

*HC* Healthy control, *AN* Anorexia nervosa, *BMI* Body mass index, *SDS* Standard deviation score, *ChEAT26* Children’s Eating Attitude Test, *AQC* Autism-Spectrum Quotient Children’s version

### Assessment measures

#### Sensory Profile (SP) and Adolescent/Adult sensory Profile (AASP)

The Sensory Profile is a standardized instrument for assessing sensory processing and profiling the impact of sensory processing on functional behavior in daily life [23–25]. Sensory processing patterns are classified into four quadrants (Low Registration, Sensation Seeking, Sensation Sensitivity, and Sensation Avoiding) based on high and low neurological thresholds for sensory stimuli, and the behavioral responses (passive and active) associated with those thresholds are scored for each quadrant. Low Registration indicates the degree to which a person misses sensory input, Sensation Seeking indicates the degree to which a person explores sensory input, Sensory Sensitivity indicates the degree to which a person detects sensory input, and Sensation Avoiding indicates the degree to which a person is bothered by sensory input [23].

The Sensory Profile is an objective scale that is assessed by the parents and other caregivers [23, 25]. The SP was developed by Dunn et al., translated into Japanese, and standardized for ages 3–82, and it is widely used in clinical settings, especially in the field of occupational therapy [25, 26]. The questionnaire consists of 125 items divided into three major categories: the sensory processing category (auditory, visual, vestibular, touch, multisensory, and oral sensory processing), which indicates the child’s responses to the basic sensory systems; the coordination category (sensory processing related to endurance/tone, modulation related to body position and movement, the modulation of movement affecting activity level, the modulation of sensory input affecting emotional responses, and the modulation of visual input affecting emotional responses and activity level), which reflects participants’ control of neurotransmissions from the facilitation or inhibition of various responses; and the behavior and emotional responses category (Emotional/Social responses, Behavioral outcomes to sensory processing, and Items indicating thresholds for response), which reflects the results of participants’ sensory processing in their behavior [25, 26]. Each item is scored on a five-point Likert scale. In this study, we examined the quadrant and section scores.

The AASP is a self-rated questionnaire that can be used to assess subjective sensory processing experiences [24, 27]. The AASP was also developed by Dunn et al. and translated into and standardized in Japanese. The AASP is a self-assessment questionnaire that can be used to assess subjective sensory processing experiences. It consists of 60 items in the following sections: Taste/Smell, Movement (Vestibular/Proprioceptive), Visual, Touch, Activity level, and Auditory processing. Each item is scored on a five-point Likert scale.

#### Children’s eating attitude test (ChEAT-26)

The ChEAT-26 is a 26-item self-administered questionnaire assessing eating attitudes and behavior [28, 29]. The child version has since been validated in Japan [30]. Each item is scored on a six-point Likert scale: “never,” “rarely,” “sometimes,” “often,” “usually,” and “always.” In the Japanese version, a score of 18 and above indicates that an individual should be considered for eating disorders, and the 26 items are divided into five subscales assessing “preoccupation with thinness”, “food preoccupation”, “dieting”, “social pressure to eat”, and “purging” [30].

#### Autism-spectrum Quotient Children’s version (AQC)

The AQC is a 50-item parent-administered questionnaire assessing autistic traits for children [31]. The Japanese version has already been validated for children between the ages of 6 and 15 years [32]. In the Japanese version, a score of 25 and above indicates that an individual should be considered for a specialist autism assessment, and this version is divided into five subscales assessing “social skills”, “attention switching”, “attention to detail”, “communication” and “imagination.” [32].

### Analysis

All data were analyzed using the IBM SPSS version 28 (Armonk, NY: IBM Corp.). The normality of data was assessed using the Shapiro–Wilk test, and normally distributed data were tested for equivariance using the Levene test. Based on the results of the normality test, comparisons between the AN and HC groups were performed with unpaired t tests for age, height, weight, BMI, and BMI-SDS and with the Mann–Whitney U test for SP/AASP, AQC, and ChEAT-26 score comparisons. Cohen’s d was used for the effect size in the t tests and Pearson product-moment correlation coefficient for the Mann Whitney U test. Spearman’s rank correlation coefficient was used for correlation analysis. For all tests, a two-tailed *p* value < 0.05 was considered statistically significant.

## Results

The demographic and background data of the participants are shown in Table 1. There was no significant difference in age between the AN group and the control group. Weight, BMI, and BMI-SDS were all significantly lower in the AN group than in the control group, a finding consistent with the diagnosis of AN.

The AQC score tended to be higher in the AN group than the control group for the total score and subscales, but only the difference in the “attention to detail” score was statistically significant. In the ChEAT, the score of the AN group was statistically significantly higher than that of the control group on all subscales except “preoccupation with thinness” and in the overall score.

Table 2 shows the quadrant and section scores on the AASP for the AN and control groups. Contrary to our hypothesis, there was no statistically significant difference between groups in the quadrant and section scores on the AASP.

**Table 2:** AASP scores

	HC group (*n* = 63)	AN group (*n* = 17)	*P*	*r*
Low Registration	29(15–53)	31(17–45)	0.92	0.01
Sensation Seeking	37(16–52)	36(27–50)	0.58	0.06
Sensory Sensitivity	30(15–51)	34(21–53)	0.14	0.17
Sensation Avoiding	30(15–52)	32(19–46)	0.30	0.12
Taste/Smell processing	16(8–26)	18(11–28)	0.10	0.18
Movement processing	16(8–26)	20(10–27)	0.05	0.22
Visual processing	22(10–33)	22(15–31)	0.13	0.17
Touch processing	26(13–46)	31(16–42)	0.09	0.19
Activity level processing	26(10–39)	24(12–29)	0.08	0.19
Auditory processing	24(11–43)	25(12–37)	0.76	0.03

Due to nonnormal distributions, statistics reported are medians followed by the range in parentheses. Pearson product-moment correlation coefficient (*r*) was used for the effect size

*HC* Healthy control, *AN* Anorexia nervosa, *AASP* Adolescent/Adult Sensory Profile

Table 3 shows the quadrant and section scores of the SP for the AN and control groups. Among the quadrant scores, only the Sensation Avoiding score was significantly higher in the AN group than the control group (*p* < 0.01, *r* = 0.35), while the Emotional/Social responses score tended to be significantly higher in the AN group, and Vision tended to be higher in the AN group, but the effect size was low.

**Table 3:** SP scores

	HC group (*n* = 63)	AN group (*n* = 17)	*P*	*r*
Low Registration	17(15–36)	17(15–51)	1.0	0.00
Sensation Seeking	26(26–47)	28(26–37)	0.09	0.19
Sensory Sensitivity	23(20–43)	23(19–38)	0.82	0.03
Sensation Avoiding	39(30–90)	52(35–93)	**< 0.01**	0.35
Auditory processing	10(8–20)	9(7–24)	0.08	0.2
Visual processing	10(9–22)	12(9–26)	**0.04**	0.23
Vestibular processing	11(11–21)	11(11–21)	0.66	0.05
Touch processing	19(18–32)	20(18–37)	0.14	0.17
Multisensory processing	7(7–17)	7(7–11)	0.34	0.04
Oral sensory processing	12(12–30)	13(12–28)	0.29	0.12
Sensory processing related to endurance/tone	10(9–25)	9(9–37)	0.83	0.02
Modulation related to body position & movement	10(10–16)	10(10–17)	0.54	0.07
Modulation of movement affecting activity level	11(7–28)	13(7–23)	0.23	0.13
Modulation of sensory input affecting emotional responses	4(4–10)	4(4–8)	0.64	0.05
Modulation of visual input affecting emotional responses and activity level	4(4–14)	4(3–10)	0.35	0.11
Emotional/Social responses	22(17–65)	36(21–56)	**< 0.01**	0.46
Behavioral outcomes of sensory processing	7(6–18)	10(6–14)	0.12	0.18
Items indicating thresholds for response	3(3–5)	3(3–4)	0.80	0.03

Due to nonnormal distributions, statistics reported are medians followed by the range in parentheses. Pearson product-moment correlation coefficient (*r*) was used for the effect size

*HC* Healthy control, *AN* Anorexia nervosa, *SP* Sensory Profile

As an exploratory study, we examined the correlations between AASP and SP quadrant scores and BMI, total ChEAT scores, and subscales (Table 4). In the AN group, AASP Sensory Sensitivity and Sensation Avoiding were significantly correlated with the ChEAT total score and the subscale scores of “preoccupation with thinness” and “food preoccupation”. Although the HC group also showed a weak correlation with “preoccupation with thinness,“ the AN group showed a stronger correlation. In contrast, in SP, Sensation Seeking was correlated with the ChEAT total score and “food preoccupation,“ “dieting,“ and “social pressure to eat”.

**Table 4:** Correlation between AASP, SP, and ChEAT26

	BMI		ChEAT26 total score		Preoccupation with thinness		Food preoccupation		Dieting		Social pressure to eat		Purging	
	CC:r	*P*	CC:r	*P*	CC:r	*P*	CC:r	*P*	CC:r	*P*	CC:r	*P*	CC:r	*P*
AASP														
HC														
Low Registration	0.29	0.02	**0.35**	**< 0.01**	**0.42**	**< 0.01**	**0.37**	**< 0.01**	− 0.10	0.42	**0.31**	**0.02**	0.10	0.45
Sensation Seeking	0.10	0.45	0.10	0.41	**0.35**	**< 0.01**	− 0.02	0.89	0.01	0.95	− 0.12	0.37	0.01	0.94
Sensory Sensitivity	0.18	0.16	0.24	0.06	**0.38**	**< 0.01**	0.15	0.26	0.03	0.82	0.03	0.79	− 0.05	0.72
Sensation Avoiding	0.18	0.16	0.15	0.05	0.25	0.05	− 0.07	0.57	0.04	0.73	0.05	0.68	− 0.17	0.17
AN														
Low Registration	− 0.03	0.90	0.42	0.10	0.42	0.09	0.40	0.11	0.25	0.33	0.32	0.22	0.35	0.17
Sensation Seeking	0.15	0.57	0.04	0.88	0.03	0.90	− 0.01	0.97	0.04	0.88	− 0.02	0.92	0.04	0.87
Sensory Sensitivity	0.16	0.53	**0.61**	**< 0.01**	**0.65**	**< 0.01**	**0.49**	**< 0.01**	0.47	0.06	0.43	0.09	0.43	0.09
Sensation Avoiding	0.24	0.36	**0.59**	**0.01**	**0.63**	**< 0.01**	**0.62**	**< 0.01**	0.44	0.08	0.31	0.22	0.39	0.22
SP														
HC														
Low Registration	0.21	0.09	0.14	0.27	0.08	0.53	− 0.01	0.92	0.05	0.69	**0.26**	**0.04**	− 0.07	0.58
Sensation Seeking	0.19	0.14	0.16	0.22	0.04	0.78	0.08	0.55	0.05	0.68	**0.28**	**0.03**	− 0.11	0.40
Sensory Sensitivity	0.18	0.16	0.08	0.53	0.14	0.27	− 0.11	0.40	0.02	0.86	0.21	0.10	− 0.13	0.32
Sensation Avoiding	0.09	0.50	0.04	0.76	− 0.04	0.79	− 0.08	0.54	0.04	0.76	0.27	0.03	− 0.01	0.96
AN														
Low Registration	0.23	0.37	0.29	0.26	0.10	0.72	0.39	0.12	0.37	0.19	0.41	0.11	0.22	0.40
Sensation Seeking	0.42	0.10	**0.59**	**0.01**	0.41	0.11	**0.50**	**0.04**	**0.58**	**0.02**	**0.58**	**0.01**	0.06	0.81
Sensory Sensitivity	0.29	0.26	0.35	0.17	0.28	0.29	**0.57**	**0.02**	0.33	0.19	**0.60**	**0.01**	− 0.01	0.77
Sensation Avoiding	0.29	0.26	0.41	0.10	0.23	0.38	0.37	0.15	0.44	0.08	0.36	0.16	**0.61**	**< 0.01**

Bold characters indicate significant statistics at *p* < 0.05

*HC* Healthy Control, *AN* Anorexia Nervosa, *AASP* Adolescent/Adult Sensory Profile

## Discussion

To the best of our knowledge, this is the first study to assess the sensory processing characteristics of children and adolescents with AN in the early and acute stages, less than one year after the onset. Based on reported previous findings, we hypothesized that children with AN in the very early stages would have altered sensory processing, similar to that of adults with AN.

However, the results did not support our hypothesis, especially in the evaluation of subjective sensory experiences. Previous reports have shown that patients with AN show sensory hypersensitivity in questionnaires of subjective sensory processing characteristics [7–9]. In addition, sensory sensitivity is reported to be associated with weight loss. Furthermore, it has been reported that hypersensitivity persists even after weight regain [7, 8]. However, in this study of children and adolescents with eating disorders in the early and acute stages, there were no findings of hypersensitivity compared to healthy control participants. On the SP, a parents’ perspective assessment, the AN group consistently showed no significant difference in sensory sensitivity scores compared to the HC group. We want to discuss the possible reasons for this finding in terms of two points.

The first point is that the timing of the present study was in the acute phase of treatment. In a previous report that evaluated sensory processing characteristics before and after acute inpatient treatment, it was reported that over-responsiveness to sensory stimuli was higher at the time of weight gain, which may have resulted in undervaluation at the time of the present study. However, in this report, the participants were over-responsive to sensory stimuli even before they gained their weight compared to healthy participants [8]. Therefore, it is unlikely that the acute stage of evaluation was the only factor in the present results.

The second point is that most of the patients participated in this study were in the first episode and very early stage of the disease, less than one year after onset, which means less affected by chronic starvation. Previous reports have focused on patients more than one year after the onset of symptoms and have not examined whether sensory responses differ in the early stage of the disease [5, 7–9]. This study is the first to report on this issue. Therefore, it is possible that the changes in sensory processing have not yet appeared in patients with very early-stage AN. On the other hand, from the parents’ perspective, there was a significant difference in the Sensation Avoiding score between groups. Furthermore, the results of the current study suggest that higher scores on Emotional/Social responses were the main factor contributing to the higher Sensory Avoiding. From the parents’ perspective, it is understood that although patients with AN are not sensitive to sensory stimuli, they have greater emotional responses when they perceive unexpected sensory stimuli. This supports previous reports that patients with AN struggle with emotional regulation [33] from a sensory processing perspective. Interestingly, from the patients’ perspective, they were not aware of their own avoiding behavior toward sensory stimuli. It is believed that the starvation associated with AN causes changes in the reward system, particularly an abnormally elevated prediction error response, which plays an important role in the learning process [34–37], and adolescents with AN have higher stimulus-response learning in both implicit and explicit learning [38]. These findings lead to the hypothesis that people with AN develop sensory hypersensitivity due to changes in the learning process caused by chronic starvation. It is possible that early in onset, patients themselves are unaware of their avoiding of unpredictable sensory stimuli, but as starvation persists, learning about sensory stimuli that are more likely to occur unpredictably is facilitated, sensory sensitivity is enhanced, and conscious avoiding of the sensation would be developed. Based on this hypothesis, hypersensitivity (lowered threshold for sensory stimuli) in AN may not be a trait symptom but rather a “scar” caused by chronic starvation. The present study was not designed to examine the relation between sensory processing and learning process changes, so we cannot discuss this hypothesis, but the relation between sensory processing and reward system changes can be an issue for future research.

In the present study, we also found correlations between Sensory Sensitivity, Sensation Avoiding and ChEAT-26 preoccupation with thinness and food preoccupation scores in adolescents with AN but not with BMI-SDS. This suggests that sensory sensitivity is related to the pathology of eating disorders and may also have a state-symptom component that is not related to weight. The finding that sensory processing problems have a state-symptom component is consistent with previous reports using physiological examinations [39]. To clarify whether these symptoms in patients with AN are traits, states, or “scars,“ larger studies with patients in the very early stages of the disease or studies with twins may be helpful [3].

It is clinically meaningful to understand the sensory processing characteristics of patients with AN. However, the SP and AASP have many items and are burdensome, making them unsuitable for longitudinal assessments. A simple method with a small number of items, such as the Brief Sensory Screener, may be used for longitudinal evaluation [9].

There are several important limitations to this study. First, the study was conducted at a single center in Japan, which may have led to selection bias. There have been no reports examining sensory processing characteristics in patients with AN in Japan. The small number of patients with AN is also an important limitation. For example, the results of the sample size calculations using the data from this pilot study indicate that a sample size of about 30–40 people could result in significant differences in AASP Sensory Sensitivity scores. Therefore, it is possible that sensory sensitivity in the AN group was underestimated. Even if the underestimation exists, the increase in AASP sensory sensitivity scores in the present study is lower than previously reported in adult cases [7]. Therefore, a longitudinal, large sample size study from early in the onset of AN would provide insight into the dynamic relation between eating disorder pathology and sensory processing.

However, the findings of the present study, which examined sensory processing characteristics in the early stages of AN, are significant in that they suggest that sensory processing characteristics may be both states and “scars” due to chronic starvation.

## Conclusion

This study examined sensory processing characteristics in children and adolescents with early-stage AN using the Sensory Profile. No significant difference in Sensory Sensitivity was found between the AN group and the HC group. The results suggest that researchers should reconsider the concept that sensory processing characteristics are trait symptoms, as previously thought. However, because of the important limitations of this study, it is not clear whether alterations in the sensory processing characteristics in AN patients are due to states, traits or “scars” due to chronic starvation.

## Data Availability

The datasets analyzed in the current study are available from the corresponding author on reasonable request.

## Abbreviations

AN: Anorexia nervosa
HC: Healthy control
SP: Sensory Profile
AASP: Adult/Adolescent Sensory Profile
ChEAT-26: Children’s Eating Attitude Test
AQC: Autism-Spectrum Quotient Children’s version
BMI: Body mass index
SDS: Standard deviation score
